# Multi-modal molecular imaging maps the correlation between tumor microenvironments and nanomedicine distribution

**DOI:** 10.7150/thno.68000

**Published:** 2022-02-14

**Authors:** Nicole Strittmatter, Jennifer I. Moss, Alan M. Race, Daniel Sutton, Jaime Rodriguez Canales, Stephanie Ling, Edmond Wong, Joanne Wilson, Aaron Smith, Colin Howes, Josephine Bunch, Simon T. Barry, Richard J.A. Goodwin, Marianne B. Ashford

**Affiliations:** 1 Imaging and Data Analytics, Clinical Pharmacology & Safety Sciences, R&D, AstraZeneca, Cambridge, UK.; 2 Department of Chemistry, Technical University of Munich, Garching, Germany.; 3 Bioscience, Discovery, Oncology R&D, AstraZeneca, Cambridge, UK.; 4 Institute of Medical Bioinformatics and Biostatistics, University of Marburg, Germany.; 5 Translational Pathology & Biomarker Analysis, Translational Medicine, R&D Oncology, AstraZeneca, Gaithersburg, MD, USA.; 6 Antibody Discovery and Protein Engineering (ADPE), R&D, AstraZeneca, Cambridge, UK.; 7 DMPK, Oncology R&D, AstraZeneca, Cambridge, UK.; 8 National Centre of Excellence in Mass Spectrometry Imaging (NiCE-MSI), National Physical Laboratory, Teddington, UK.; 9 Institute of Infection, Immunity and Inflammation, College of Medical, Veterinary and Life Sciences, University of Glasgow, Glasgow, UK.; 10 Advanced Drug Delivery, Pharmaceutical Sciences, R&D, AstraZeneca, Macclesfield UK.

**Keywords:** nanomedicine, mass spectrometry imaging, tumor distribution

## Abstract

Gaining insight into the heterogeneity of nanoparticle drug distribution within tumors would improve both design and clinical translation of nanomedicines. There is little data showing the spatio-temporal behavior of nanomedicines in tissues as current methods are not able to provide a comprehensive view of the nanomedicine distribution, released drug or its effects in the context of a complex tissue microenvironment.

**Methods:** A new experimental approach which integrates the molecular imaging and bioanalytical technologies MSI and IMC was developed to determine the biodistribution of total drug and drug metabolite delivered via PLA-PEG nanoparticles and to overlay this with imaging of the nanomedicine in the context of detailed tumor microenvironment markers. This was used to assess the nanomedicine AZD2811 in animals bearing three different pre-clinical PDX tumors.

**Results:** This new approach delivered new insights into the nanoparticle/drug biodistribution. Mass spectrometry imaging was able to differentiate the tumor distribution of co-dosed deuterated non-nanoparticle-formulated free drug alongside the nanoparticle-formulated drug by directly visualizing both delivery approaches within the same animal or tissue. While the IV delivered free drug was uniformly distributed, the nanomedicine delivered drug was heterogeneous. By staining for multiple biomarkers of the tumor microenvironment on the same tumor sections using imaging mass cytometry, co-registering and integrating data from both imaging modalities it was possible to determine the features in regions with highest nanomedicine distribution. Nanomedicine delivered drug was associated with regions higher in macrophages, as well as more stromal regions of the tumor. Such a comparison of complementary molecular data allows delineation of drug abundance in individual cell types and in stroma.

**Conclusions:** This multi-modal imaging solution offers researchers a better understanding of drug and nanocarrier distribution in complex tissues and enables data-driven drug carrier design.

## Introduction

Nanomedicines can aid the development of successful new drug products by changing distribution and improving tolerability and/or increasing the drug concentration at tumor sites relative to normal tissue 1, 2. Despite this, their utility within the field of cancer treatment has been marked by a lack of successful clinical translation. We therefore need to challenge how we perform nanomedicine research and development to unlock their potential for delivery of advanced therapies. This requires new approaches in understanding their delivery but also methods to better contextualize their distribution in target tissues.

Disease heterogeneity is deemed a driving factor for poor clinical translation of nanomedicines. Disease models that are more representative of the clinical situation should therefore aid nanomedicine translation. Patient-derived tumor xenografts (PDXs), where tumor fragments are obtained from cancer patients and transplanted into immunodeficient mice, have emerged as a useful model for translational research and are better predictors of clinical efficacy 3, 4. A major advantage of PDX models is that they maintain the tumor architecture and as a result the tumor microenvironment has been shown to mimic the patient pathohistological and genetic features 5. This more closely re-capitulates the clinical situation and makes PDX models useful for understanding the accumulation, distribution and retention of nanoparticles (NPs).

New molecular imaging technologies, like mass spectrometry imaging (MSI), are offering new ways to view the tissue microenvironment that move us away from a simple targeted morphological assessment of the sample towards a view that allows assessment of exogenous compounds against a systems-level metabolic backdrop. This label-free molecular imaging can be exploited to provide enhanced information to design and aid clinical translation 6. MSI enables the spatial mapping of the local distribution of drugs alongside drug metabolites as well as endogenous tissue constituents, ranging from small metabolites 7, 8, peptides and proteins 9-12 to glycans 13. It has further been demonstrated that the endogenous metabolite profile can be used to differentiate tissue types and individual tissue features 14, 15.

To further enhance our understanding of where drug is distributed and how this relates to nanomedicine localization and target engagement requires an even more expanded multimodal imaging approach 6, 16. Imaging mass cytometry is a new technology allowing highly multi-plexed immunohistochemistry-based staining on a single tissue section. It has proved to be a powerful tool to study the tumor microenvironment and the distribution and abundance of local cell phenotypes, especially the composition and distribution of the immune subpopulations in tumors 17-19. Here we demonstrate that an integrated multi-modal imaging approach that combines MSI and IMC generates a wealth of in-depth information from the same tissue section to support the understanding of nanomedicine-mediated drug distribution and the features in tumor with which the drug and nanomedicine associate.

The ability for polymeric nanoparticles to prolong circulation and extend active drug exposure at target sites and thereby change the safety and efficacy profile of a drug is well-documented 20-23. The efficacy of anti-cancer nanomedicines is influenced by tumor morphology and the tumor microenvironment (TME) accumulation, distribution and retention of the carrier, the release of the drug and target engagement in the tumor 24. Hence many factors generate a multi-parametric space that is challenging to fully analyze. We exemplify the utility of holistic molecular imaging for an aurora kinase B inhibitor, AZD2811, NP formulation. In AZD2811-NP, the active drug from the phosphate prodrug barasertib is encapsulated in a slow-release formulation of polylactic acid polyethylene glycol (PLA-PEG) NP using an ion-pairing approach 25 which has been subsequently developed for clinical use. Treatment with AZD2811-NP led to improved efficacy and toxicity in pre-clinical models of small cell lung cancer (SCLC) 26, diffuse large B-cell lymphoma 1 and acute myeloid leukemia (AML) 27 compared to barasertib (AZD1152). Following a successful Phase I trial 28, AZD2811-NP is being explored in a Phase II study in small cell lung cancer in combination with durvalumab (NCT04745689).

MSI is increasingly gaining traction as a tool to study nanomedicine delivery and support nanomedicine development 29-32. We have previously reported the use of MSI to support the development of NP-formulated AZD2811 showing extended retention and heterogeneous localization of drug alongside drug metabolite and nanocarrier 1, demonstrating that MSI is a suitable technology to study AZD2811-NP distribution. The quantitation of AZD2811 has also been shown via MSI in biopsy specimen 33. However, to truly enhance the field of nanomedicine research, we have combined the molecular insights achieved using MSI with advanced multi-modal imaging techniques that unlock the heterogeneity of the tumor microenvironment through high-plex IHC on the same tissue samples in three patient-derived xenograft models (PDXs) with different tumor phenotypes ranging from low to high stroma content. Novel image and data analysis is now able to correlate differential distribution of NP-formulated drug versus non-NP-formulated drug across the complex TME of PDXs. This provides a far greater level of understanding of drug distribution in relation to different tumor features and cell types than previously achievable.

This exemplified research will directly support the nanomedicine field but is equally impactful for researchers working with complex and emerging drug modalities. We believe that a complete and holistic view of our tumor samples is the only way we can rapidly deliver advanced new medicines.

## Methods

### Compounds

AZD2811, formerly designated AZD1152 hydroxy-quinazoline pyrazole anilide or AZD1152-hQPA, (2-[3-[[7-[3-[ethyl(2-hydroxyethyl)amino]propoxy]quinazolin-4-yl]amino]-1H-pyrazol-5yl]-N-(3-fluorophenyl)acetamide), is an anhydrous free base and was prepared in-house (AstraZeneca, Macclesfield, UK). NPs loaded with AZD2811 (AZD2811-NP) were manufactured and characterized by BIND Therapeutics (Cambridge, MA 02139 USA) using the modified oil in water (o/w) emulsification method described previously 20. Briefly, the NPs are composed of AZD2811 (17%) incorporating a pamoic acid counterion and PLA-PEG copolymer. Characterization is described previously 25. The NP diameter is 88 nm with a polydispersity index of 0.1. Deuterated [^2^H_5_]-AZD2811 (batch AZ11792866-019) was prepared by Key Organics (Camelford, UK).

A structural analogue prepared in-house ((2-(3-((7-(3-(propyl(2-hydroxyethyl)amino)propoxy)quinazolin-4-yl)amino)-1H-pyrazol-5-yl)-N-(3-fluorophenyl)acetamide) was used for correcting variations in signal of both AZD2811 and [^2^H_5_]-AZD2811 in the different tissue matrices in DESI-MSI studies. Structures of all compounds are displayed in Figure S1.

### *In vivo* PDX study

All animal studies were conducted at Oncotest GmbH (Charles River Laboratories) in accordance with local authorities, guidelines of German Animal Welfare Act, and the AstraZeneca Global Bioethics policy. The experiments described in this article were conducted in female NMRI nu/nu mice (Harlan) delivered at 4-6 weeks of age. Mice were housed in individually ventilated cages (TECHNIPLAST), on a 14 h/10 h light/dark cycle at 25 °C +/- 1 °C with humidity maintained at 45-65%. Animals had access to food and water *ad libitum*. During the studies, mice were monitored at least daily.

*In vivo* studies were completed using three patient-derived explant (PDX) models: CXF1297 (colon adenocarcinoma), LXFE2257 (primary lung squamous cell carcinoma) and OVXF899 (primary ovary serous adenocarcinoma), herein referred to as colon, lung, and ovarian models, respectively. These models are established in female mice and were chosen due to their different characteristics in both stroma and tumor morphology. See Table S1 for more information on these three PDXs. For studies described here, tumor fragments were implanted under isoflurane anesthesia. Mice received a unilateral, subcutaneous implant into the left flank.

When tumor volumes reached 300-600 mm^3^, mice (n = 21/model) were randomized for treatment. All mice were administered AZD2811 NP-formulation at 25 mg/kg intravenously at 0.1 mL/10 g bodyweight on day 1 and day 3. At 4 h before their terminal time point, all mice were administered a single dose of [^2^H_5_]-AZD2811 at 5 mg/kg intravenously at 0.1 mL/10 g bodyweight. Tissues and plasma were collected at 7 sampling time points (n = 3 mice/time point): 4, 8, 12, 24, 72, 168 and 240 h after the second dose of AZD2811-NP. Terminal blood was collected via cardiac puncture into cold Li-Hep tubes; plasma was collected and stored at -80 °C. Tissues (tumor, muscle, spleen, liver, duodenum) were halved and snap-frozen in liquid nitrogen.

### Bioanalysis

Each plasma sample (25 µL) was prepared using an appropriate dilution factor and compared against an 11-point standard calibration curve (1-10000 nM) prepared in DMSO and spiked into blank plasma. Tumor or tissue was weighed into fast preparation tubes containing Lysing Matrix A (MP Biomedicals UK). Water is added as a base for homogenization (5 times *w*/*v*). Homogenization is carried out in FastPrep-24 5G (MP Biomedicals USA) at 6 m/s for 45 s. To both tumor and plasma samples, acetonitrile (100 µL) was added with the internal standard, followed by centrifugation at 3000 rpm for 10 min. Supernatant (50 µL) was then diluted in 300 µL water and analyzed via UPLC-MS/MS (see Table S2 and S3 for instrument details).

Each tumor homogenate sample (25 µL) was compared against an 11-point standard calibration curve (1-10000 nM; Figure S2) prepared in DMSO and spiked into blank tumor or tissue homogenate. Detected levels correspond to total drug amount (i.e. encapsulated plus released AZD2811) in the tissue sample.

### Mass spectrometry imaging

Tumors were embedded into a 2% CMC, 1% gelatin hydrogel for simultaneous processing. Fresh frozen tissues were cryosectioned to 10 µm thickness using a Leica CM3050S cryomicrotome (Wetzlar, Germany). Data were recorded using a desorption electrospray ionization mass spectrometry imaging (DESI-MSI) system consisting of a Omnispray 2D DESI source (Prosolia Inc, Indianapolis, IN, USA) and a Q-Exactive mass spectrometer (Thermo Scientific, Bremen, Germany) equipped with a home-built sprayer as described previously 34, 35. Methanol/Water (95:5 *v*/*v*) was used as electrospray solvent at a flow rate of 1.0 µL/min delivered using a Dionex Ultimate 3000 nLC pump (Sunnyvale, CA, USA). Data was recorded using a spatial resolution of 50 µm in x-direction and 75 µm in y-direction, negative ion mode and using a mass range of *m*/*z* 250-1000. More details can be found in Table S4. Imaging data were converted into mzML format using MSIConvert tool from the ProteoWizard 3.0.4043 toolbox 36 and subsequently converted into .imzML format using imzMLConverter v1.3 37.

Drug detected is always total drug, in this manuscript AZD2811 is total drug detected which was dosed as NP-formulation (i.e. encapsulated plus released AZD2811), while [^2^H_5_]-AZD2811 is total drug detected which was dosed as free drug.

### Morphological tissue classification

An adjacent tissue section was H&E stained and scanned at 40× (Hamamatsu NanoZoomer). The images were loaded into HALO v3.2 for tissue classification. For each tumor type a MiniNet Deep Learning model was trained to segment tumor, stroma and necrotic tissue. Representative areas on the slide were annotated to train the model. An example of the H&E images and corresponding tissue classification can be found in Figure S3.

### Imaging mass cytometry

Imaging mass cytometry (IMC) was performed on the same slide following DESI-MSI analysis. Compatibility of the DESI-MSI step with subsequent IMC step is shown in Figure S4. No loss of specificity or marked reduction in signal intensity was observed for any of the markers studied. After selection of regions of interest, IMC was performed using the Hyperion Imaging System (Fluidigm Corporation) at a laser power of 6 db and 200 Hz repetition rate. IMC regions were chosen based on DESI-MSI data of the same slide to contain heterogenous distribution of NP-delivered AZD2811 (areas of both high and low AZD2811 content, see Figure S5). Antibody panel applied can be found in Table 1, summary of procedure for custom antibody labelling and antibody staining can be found in the Supplementary Material. To be included into our in-house antibody library, all antibodies are validated in-house on different tissues (human and mouse origin tumor, murine liver, spleen, brain) or isolated cell populations when necessary, using conventional immunohistochemistry followed by the metal-labelling procedure and the corresponding IMC staining. The specificity of each stain was assessed in all resulting images by a trained veterinary pathologist and only those that showed sufficient specificity in both the standard IHC as well as the IMC images were used. Specificity of all stains was again assessed in the final IMC images and in comparison to adjacent section H&E images.

Image generation was performed using MCD Viewer (Fluidigm, version 1.0.560.2). Manual thresholding was performed to remove background signal, as assessed morphologically. For all comparative images, signal thresholds were set identically for each marker between samples to allow for unbiased comparison.

### Cell segmentation

Images were exported as 32-bit.ome.tiff from MCD Viewer and imported into Halo v3.1 (Indica Labs). Data was analyzed using the HighPlex FL v3 module. Cell segmentation and thresholds for individual marker positivity were set manually based on visual inspection. To further classify the immune sub-cell types we used the following phenotypes: macrophages - CD45+ CD11b+ F4/80+ or CD68+ CD11c- Ly6G-, M1-type macrophages - CD45+ CD11b+ F4/80+ or CD68+ MHCII+, phagocytotic (M2-type) macrophages - CD45+ CD11b+ F4/80+ or CD68+ CD163+ CD206+, immature monocytes - CD45+ CD11b+ F4/80+ or CD68+ Ly6G+ CD11c-, dendritic cells - CD45+ CD11b+ F4/80+ or CD68+ CD11c+, neutrophils - CD45+ CD11b+ F4/80- CD68- Ly6G+. Cell object data were exported into .csv format for cell distribution to MS image co-registration. Tissue types were classified on a per model basis using a random forest classifier into tumor, necrosis and connective tissue. Number of % positive cells over the entire ROI and tumor tissue only was exported as .csv to study morphological differences between the different models.

### MSI and IMC co-registration and correlation analysis

MSI data were preprocessed using SpectralAnalysis (to remove background regions, reduce to detected peaks and normalization) 38. Representative images were generated for both modalities and multimodal registration was performed 39. Cell data (IMC) were then transformed to the MSI space, enabling correlation analysis between IMC markers and MSI ion images. Details of the preprocessing, representative images and registration process can be found in the Supporting Information.

### Classifying MSI data using IMC data

To classify individual MSI spectra (each pixel) into one of three categories (tumor, connective tissue and necrosis), the classified cell segmented IMC data were used. Using the transformed cell data in the MSI space, MSI pixels, in which over 50% of the detected cells were assigned to a given category, were assigned the same category. A *k*-nearest neighbor classifier was then fit (with neighbors = 30) to all MSI pixels which had been assigned one of the categories (with a background category also included, comprised of pixels from the 'background' determined by *k*-means). Prediction was then performed on the entire MSI dataset, including regions not analyzed by IMC. Resulting tissue classes in tumor and corresponding training regions can be seen in Figure S6. Mean ion intensities for selected ions of interest were generated on a per tissue basis for each of the categories.

## Results

### Morphological tissue characterization of PDX tumors

**Figure 1:**
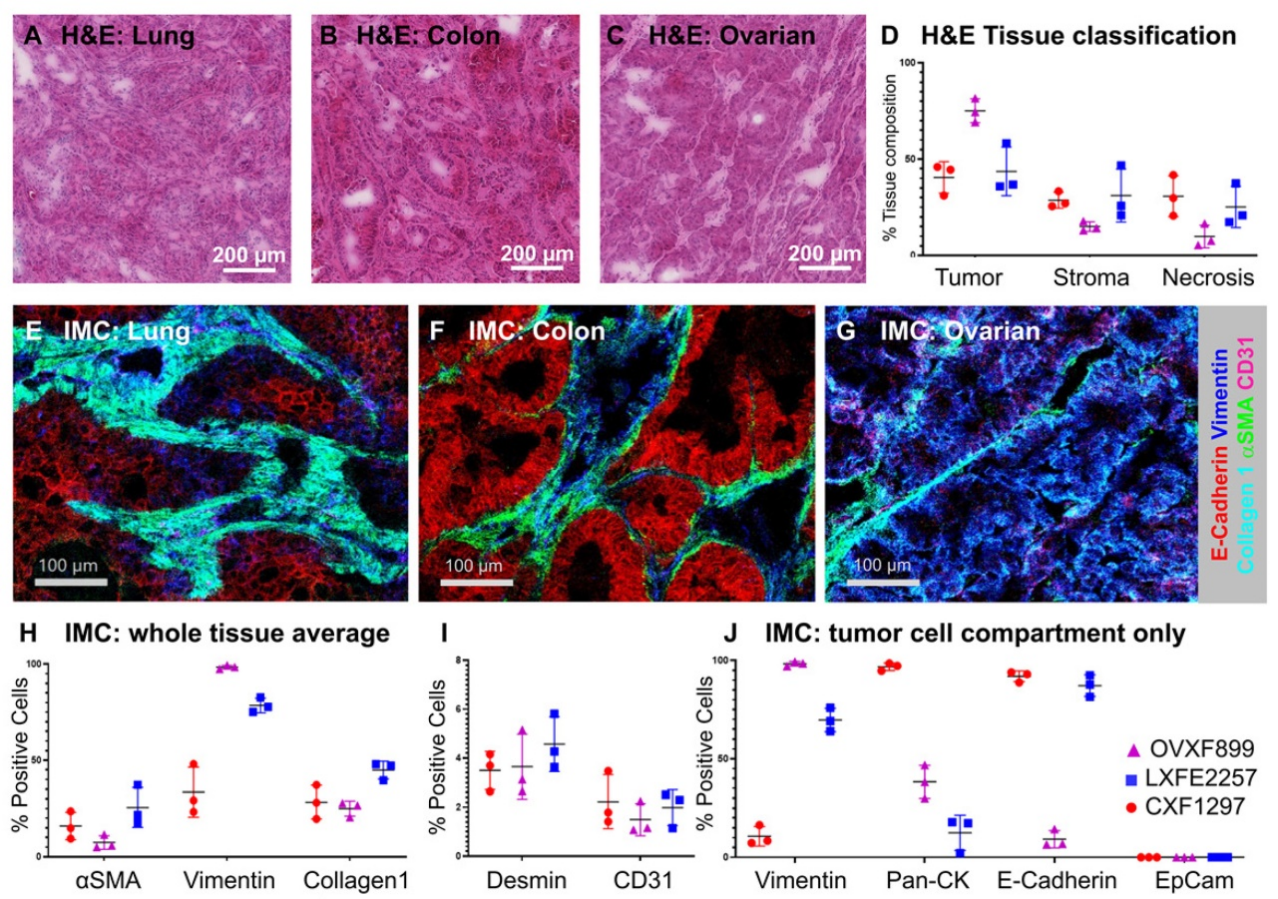
** A-C)** H&E images of A) lung (LXFE2257), B) colon (CXF1297), C) ovarian (OVXF899) PDX models; **D)** Tissue composition of H&E data derived using Halo AI classifier; **E-G)** Close-up of tissue morphology for each model based on IMC data: E-Cadherin (red), vimentin (blue), collagen 1 (cyan), αSMA (green), CD31 (pink), for E) lung, F) colon and G) ovarian model, respectively. **H & I)** Percentage positive cells over whole tissue area and J) tumor cell compartment only for morphological tissue markers for each PDX model (n=3 each) by IMC. Location of ROI for each tissue section used for H-J indicated in MSI and H&E image shown in Figure S6 and S7, respectively.

Assessing local distribution of nanomedicines in the tumor has been a challenge in the field for many years. These insights are critical to answer key questions such as features that influence distribution and retention of particle and drug in the tumor. To analyze the distribution of the PLA-PEG-based AZD2811 NP in a complex tumor microenvironment three different human PDX models (ovarian, lung and colon) with heterogeneous tumor cell, tumor-associated stroma including collagen fibers and blood vessel architecture were selected. H&E (Figure 1A-D) and IMC analysis (Figure 1E-J) was used to examine differences in the key biomarkers in the three models. Similar amounts of tumor and stroma were present in the lung (average of 44% tumor and 31% stroma) and colon PDX models (41% tumor and 29% stroma), while the ovarian tumors showed a higher percentage of tumor and a lower percentage of stroma compared to the other two models (75% and 15% respectively, Figure 1D).

The three PDX models had different stromal architecture. The lung model was characterized by a densely organized αSMA-positive stroma (fibroblasts/pericytes), the colon model was organized but less dense, while the ovarian had less dense stromal content (Figure 1E-G). Each model had similar amounts of vasculature, although the distribution was different between models. On the epithelial-mesenchymal axis, the ovarian model presents as the most mesenchymal while the colon model presents as the most epithelial (Figure 1J).

### Biodistribution of AZD2811 in three PDX models analyzed via LC-MS

**Figure 2:**
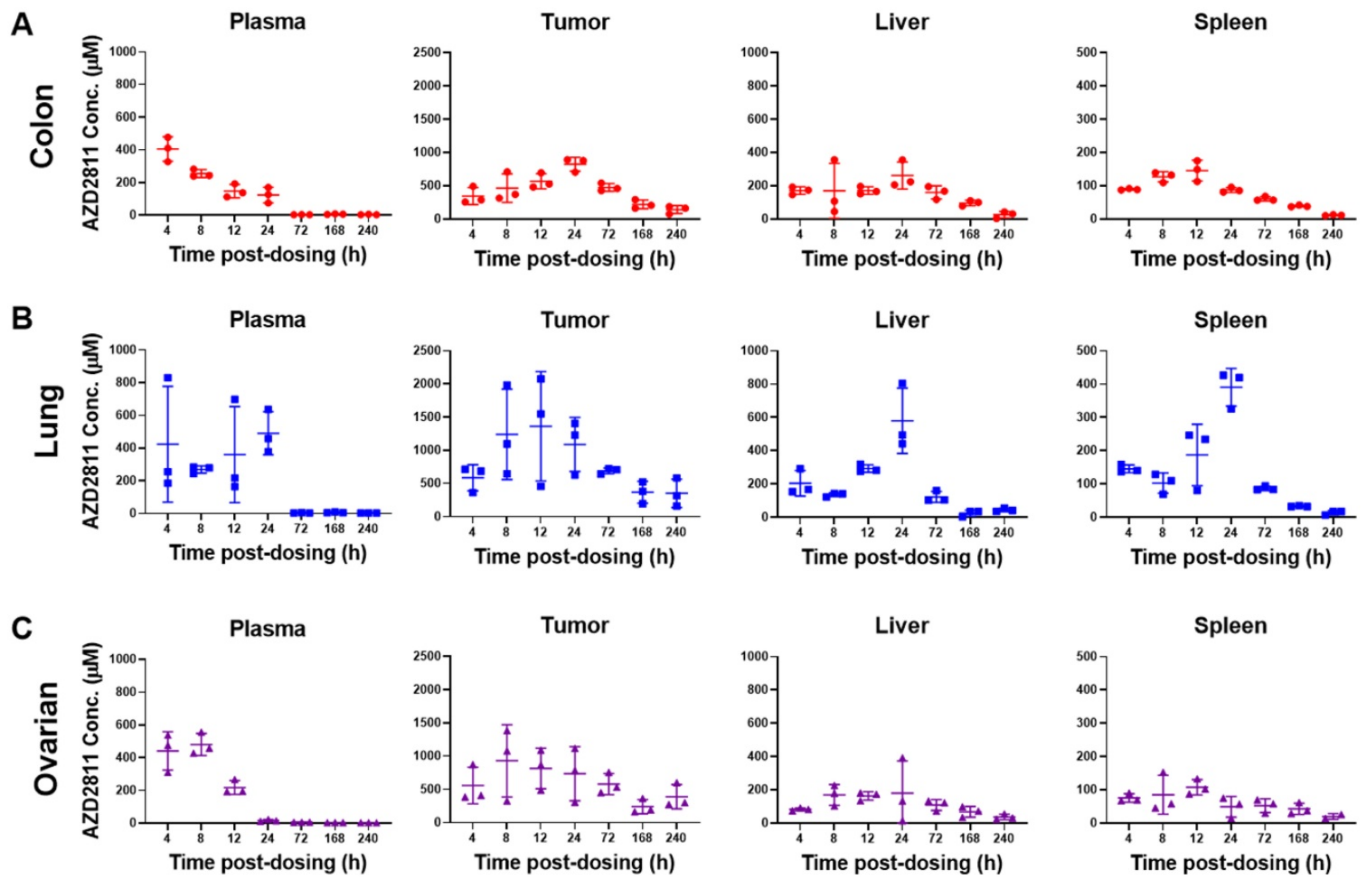
Concentration of AZD2811 determined using LC-MS/MS in plasma, tumor, liver and spleen at time points between 4 and 240 h post-second dose of AZD2811-NP at 25 mg/kg IV to mice (n = 3 mice/time point) bearing colon cancer (CXF1297; **A**), non-small cell lung cancer (LXFE2257; **B**), or ovarian cancer (OVFX899; **C**) PDX tumors.

To assess the whole tissue biodistribution of AZD2811-NP, classical LC-MS techniques were used. Total AZD2811 concentration was measured in plasma and tumor, as well as in the clearance organs liver and spleen (see Figure 2 and S8A). In addition, concentration profile for muscle and duodenum are displayed in Figure S8B. Low levels of the AZD2811 metabolite were also detected post-dose in plasma and tissues in all three PDX models (Figure S8C).

Administration of the AZD2811-NP formulation prolongs drug exposure in plasma and organs relative to the administration of free [^2^H_5_]-AZD2811 1. NP-formulated AZD2811 was administered on day 1 and day 3, and the time course following the second dose was analyzed. Concentrations of AZD2811 in plasma, tumor, and key tissues were similar between mice bearing the three PDX tumors (Figure 2 and S8A). C_max_ in plasma occurred at the time of IV dosing and plasma concentration was undetectable at 72 h, while the C_max_ of total AZD2811 in tumor and liver was observed at ~24 h. The biological reason for the larger variation in the lung PDX tumor is not clear, but is believed to represent true variation in the biodistribution of the NP in these mice bearing the lung PDX tumors. Tumor concentration of AZD2811 was measured up to 10 days after dosing (161-316 µM), supportive of NP retention (Figure 2). This biodistribution pattern and tumor retention is consistent with previous work in SW620 tumor xenografts 1.

When normalized for organ weight, the percent injected dose of AZD2811 per gram (% ID/g) was consistent between models following 2 doses of AZD2811-NP (Table S6). The highest % ID/g was in liver (11-15% ID/g), approximately double the % ID/g in spleen. The % ID/g in tumor was 9.2% ID/g for the colon model, 8.0% ID/g for the lung model, and was slightly lower in the ovarian model at 6.1% ID/g. These data are slightly higher than published data for PLA-PEG NP biodistribution in MX-1 mouse breast xenograft tumors, where 4.7% ID/g was observed at 48 h post-dose 40. Overall, the AZD2811-NP distribution was largely consistent between animals bearing different PDX tumors.

### MSI of NP formulation vs IV formulation (AZD2811 vs [^2^H_5_]-AZD2811)

**Figure 3:**
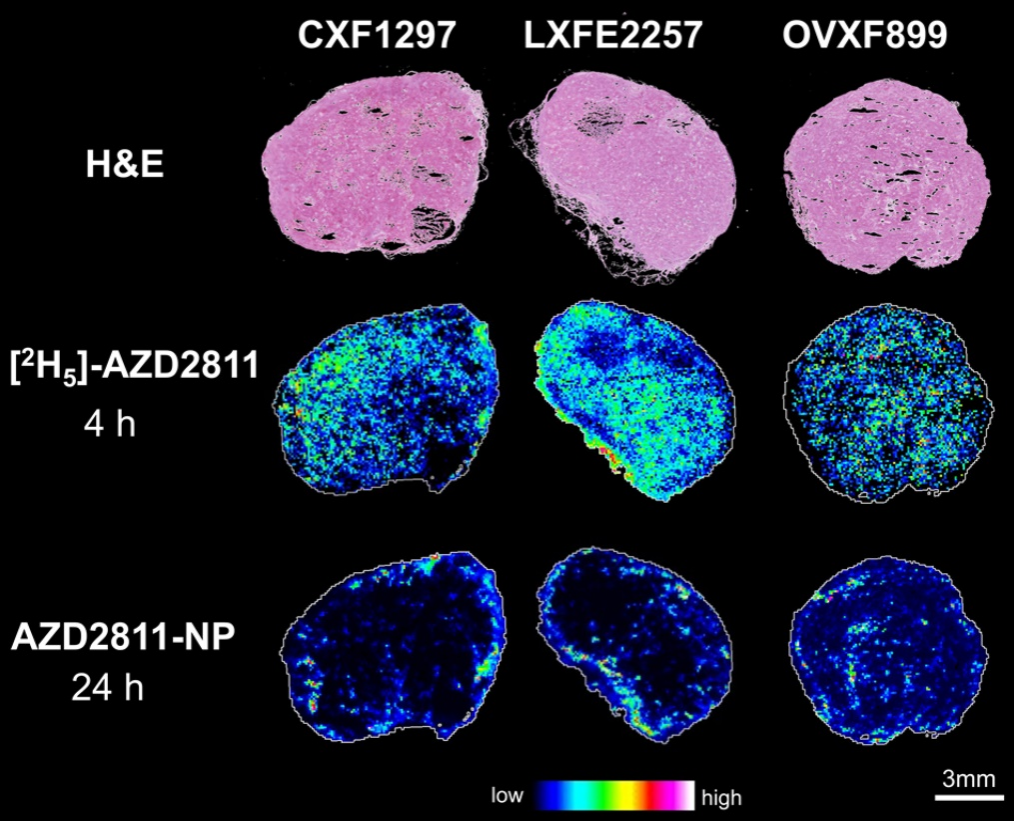
H&E images and distribution of free [^2^H_5_]-AZD2811 dosed at 4 h and NP-delivered AZD2811 at 24 h post-dose for colon (CXF1297), lung (LXFE2257) and ovarian (OVXF899) PDX models (both time points correspond to the respective C_max_). Both drug images were recorded by DESI-MSI and normalized to *m*/*z* 520.2492 ± 0.005 (structural analogue).

Nanomedicine formulations are used to change the distribution and PK profile of drugs. However, analysis of the concentration of drug in bulk tissue does not describe the local-regional distribution of drug in tumor or tissues. To investigate heterogeneity of AZD2811 delivered via NP versus free drug distribution in the tumor, a novel MSI-based imaging approach was used. This strategy has been used to visualize differences in the distribution of salmeterol in lung by co-dosing inhaled salmeterol and intravenous deuterated [^2^H_3_]-salmeterol 41. A modified version of this technique was used to directly visualize the effect NP has on the drug distribution profile within the tumors. Free [^2^H_5_]-AZD2811 and AZD2811-NP were co-dosed and the distributions were compared by MSI. This enables, for the first time, to directly compare the distribution of NP-delivered and free drug in the same tumor and the same tissue section. The intra-tumoral distribution of AZD2811 delivered via the NP and IV [^2^H_5_]-AZD2811 was clearly distinct at both the same (see Figure S9) and the respective C_max_ time point (Figure 3). Distribution images of both compounds in liver and spleen can be found in Figure S10. A homogenous distribution of free, deuterated drug can be observed in all three tumor models with comparable levels of drug in the tumor core and the tumor periphery. In contrast, in the same tumor slice, distribution of AZD2811 delivered via the NP is heterogenous with high levels of drug observed in the periphery of the tumor tissue. Higher relative distribution into the tumor core was observed in the ovarian tumor model, however, regions of high and low drug distribution remain visible. This approach delivers new insight into the behavior of the PLA-PEG nanomedicine, but the approach is directly transferable to other NP formulations.

### Correlation of the TME with AZD2811 distribution

**Figure 4:**
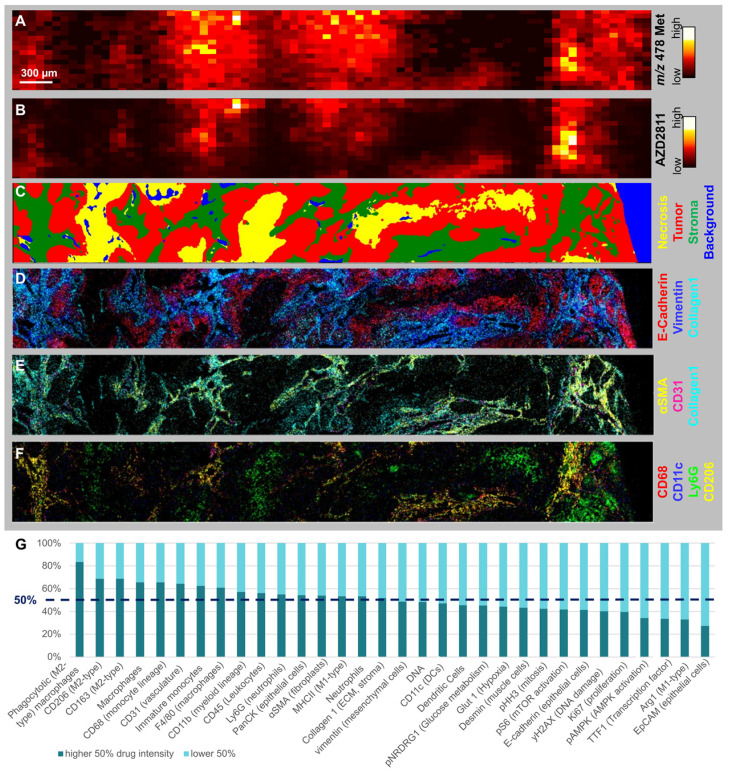
** Assessing the spatial distribution of drug metabolites, the release of drug from the NP and relating to TME (A-B) MSI and C-F) IMC images of lung tumor 24 h specimen. A)** Distribution of AZD2811 Hydroxy-QPA-N-desethyl metabolite (detected at *m*/*z* 478) and **B)** AZD2811 by DESI-MSI. **C-G)** IMC data of same tissue area: **C)** Random Forest tissue classification model. **D)** Markers displaying basic tumor architecture: E-cadherin (epithelial cells), Vimentin (mesenchymal cells), collagen1 (ECM, stroma). **E)** Markers displaying the perivascular space: collagen 1, αSMA (fibroblasts), CD31 (vasculature). **F)** Markers showing key myeloid populations (CD68 macrophages, CD68+CD11c dendritic cells, Ly6G neutrophils, CD68+CD206 phagocytotic (M2-type) macrophage subtype). G) Stacked bar graph showing abundance of cell markers in stroma with high vs. low drug content for same tissue.

Few studies have been able to robustly determine the relative distribution of nanomedicine and drug between the stroma and tumor cell compartments. While NP-encapsulated versus free drug cannot be quantified directly, drug release can be inferred by visualizing the spatial distribution of drug metabolites (Figure 4A) 1. The AZD2811 metabolite N-Desethyl-hydroxy-QPA was diffusely distributed around the areas with high AZD2811 abundance (Figure 4B), implying drug is released from the NP and locally metabolized rather than originating from peripheral circulation. To investigate what tissue features are associated with high and low concentrations of AZD2811-NP, biomarkers of tumor and TME visualized by IMC were assessed in the regions showing high and low concentrations of AZD2811. For all tumors, drug abundance correlated with stroma and macrophage populations (Figure S10 and Table S7 for Pearson correlations), exemplified in detail for the 24 h timepoint in the lung PDX model (Figure 4). A comparison of the spatial distribution of AZD2811-NP, metabolite and IMC tissue classification for all three PDX models for colon and ovarian at 24h is shown in Figure S11. Similar analysis as shown in Figure S7 was not applicable for the drug metabolite due to the high variability in metabolite abundance which resulted in correlation values that were not comparable between tissues. The AZD2811 distribution (Figure 4B) shows good spatial correlation with the tumor stroma compartment (Figure 4C). Distribution of the NP-delivered drug to the stroma likely reflects that these regions tend to have higher vessel density in the three PDX tumor models used here. AZD2811 also co-localizes with stromal markers vimentin, Collagen 1 and αSMA, vasculature marker CD31, macrophage markers CD11c, CD68 and CD206, but less so for the neutrophil marker Ly6G (Figure 4D, E and F).

Some studies report that NPs are preferentially taken up by immune cells in the TME which can then act as drug depots 42, 43. Other cell types present in the TME may influence nanomedicine distribution or be impacted by cancer nanomedicines. The methodology presented herein can be expanded to look at additional markers of interest and answer specific questions as required. Further studies are needed to assess whether AZD2811 is enriched in immune cells, or whether NPs tend to associate in regions that are more accessible, and coincidentally have higher levels of recruited immune cells. It would be interesting to determine whether the distribution of AZD2811, and other cancer nanomedicines, to macrophage-rich regions modifies the behavior of tumor macrophages; this could be achieved by using a bespoke macrophage phenotyping panel in multi-modal analysis.

Interestingly, even within the stromal compartment, AZD2811 distribution was heterogenous (see Figure S12). To gain further insight into this heterogeneity, the correlation with stromal features in areas of higher versus lower AZD2811 abundance were studied. In the lung tumor section at 24 h, this revealed that stromal regions with higher AZD2811 abundance were associated with a 5.1-fold higher accumulation in phagocytotic M2-type macrophages (CD45+ CD11b+ F4/80+/CD68+ CD206+ CD163+) as well as a 1.8-fold higher occurrence of CD31+ (vasculature marker) endothelial cells (Figure 4G).

**Figure 5:**
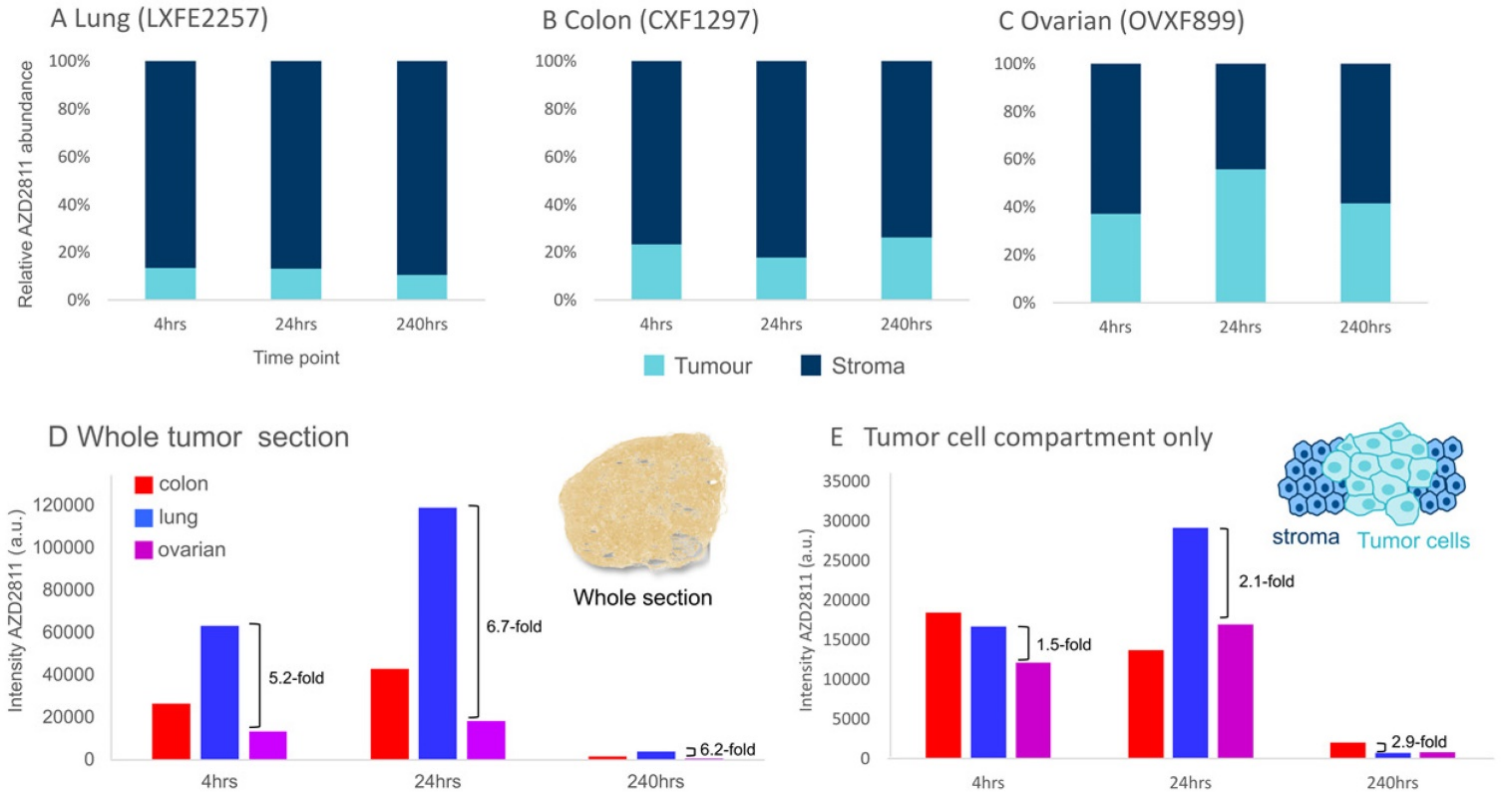
** Assessment of the model-dependent percentage of drug in the tumor compartment. (A-C)** Relative amount of NP-delivered AZD2811 in the tumor vs stroma tissue compartment normalized to the total amount of drug in each section as determined using DESI-MSI. **D & E)** Average amount of NP-delivered drug (AZD2811) over the entire tissue section (D) and the tumor cell compartment only (E).

The MSI-IMC imaging protocol can be applied to analyze relative distribution of drug to specific biomarker-defined regions. This was deployed to determine the relative amount of AZD2811 in tumor vs stroma over time for each model to investigate whether distribution ratios into the tumor compartment change over the investigated time course. The amounts of AZD2811 detected in the tumor and stroma were normalized to the total amount of drug in the tissue. As Figure 5A-C show, a different relative amount of AZD2811 was present in the tumor compartment in each model, however, no changes to the relative amount were observed over time. In lung and colon PDXs, the two high stroma models, the amount was lower, approximately 10% for lung, 20% for colon, while the low stroma ovarian model had approximately 45% total drug distributed to the tumor compartment. This may imply that while the total amount of drug is highest in lung, followed by colon and lowest in the ovarian PDX (see Figure 5D), these initial differences in drug uptake into the tumor in the lung versus ovarian PDX decrease 2-3-fold (from 5.2-6.7-fold difference for the whole tumor to 1.5-2.9-fold) when assessing the tumor cell compartment alone.

## Discussion

As the TME is thought to influence tumor accumulation, distribution and retention of nanomedicines, visualizing the distribution of NP-delivered drug more accurately in tumors will aid their design and development. Classically, biodistribution analysis has been performed at the bulk tissue level using radioactively labelled drugs. To date, a key limitation has been the ability to produce high quality simultaneous visualization of drug as well as relevant tissue features at scale. Several groups have combined pharmacokinetic measurements with optical imaging to investigate NP uptake and cellular distribution in tumors using standard imaging techniques or complex visualization techniques like intra-vital microscopy 42, 43. However, ambiguity remains as fluorescent labelling of the NPs may alter their physico-chemical properties or the tumor model or tumor tissue used in the assay may not reflect the clinical situation. Thus, in addition to the standard LC-MS-based bioanalysis, we propose a multi-modal imaging workflow to study the detailed distribution of a clinical stage PLA-PEG NP (AZD2811-NP) in three PDX tumor models with differing TME features more reflective of patient tumors 44.

Prolonged plasma circulation time has been shown to be critical for good tumor accumulation of nanomedicines 45, 46. The AZD2811 exposure in plasma and tissues was as expected 1, and similar to other PLA-PEG NPs with a tumor C_max_ at 24 h 20, 40. The pharmacokinetic profile of AZD2811-NP over time was largely consistent between models. The% ID/g of tumor was in the 6-10% range in all PDX tumors. This is consistent with previous studies on the same NP and significantly greater than many of the early first generation NPs where less than 1% ID/g reached the tumor 47. It can however be challenging to understand the difference in tumor distribution between NP-delivered drug and free drug reaching the tumor from peripheral drug exposure. Using MSI, intra-tumoral distribution of NP-delivered AZD2811 and the deuterated free drug at the C_max_ time point after intravenous dosing were readily co-visualized, enabling differential drug distribution to be directly characterized in the same tissue. While the distribution of [^2^H_5_]-AZD2811 was uniform across each tumor section, the NP-delivered AZD2811 exhibited heterogeneous distribution. Studies on similar sized NPs using other imaging modalities have also shown heterogeneous distribution of the NPs in the tumor, with higher concentration around the periphery as seen here (Figure 3) 4, 48.

Differences in distribution between tumors were seen in the stroma-rich lung and colon models versus the stroma-low ovarian model. Being able to couple MSI and IMC analysis enabled detailed definition of the ratio of drug distribution into specific regions of the tumor, and determination of features associated with both high and low drug abundance. Interestingly, although the stroma-rich lung and colon models exhibited greater whole tumor drug concentrations in both MSI and LC-MS bioanalysis, the highest drug abundance in these tissues was localized to the stroma rather than the tumor cell compartment. In contrast, while the low-stroma ovarian model had the lowest bulk tumor AZD2811 accumulation of the three models, proportionally there was greater AZD2811 present in the tumor compartment. This suggests lower NP accumulation and retention in these tumors may be offset by more efficient distribution. Very detailed assessment of NP and tissue biomarker distribution in 3D in individual tumors could be amassed by analyzing multiple slices throughout the tumor.

These insights have interesting implications. While the stroma-enriched TME may appear to reduce access of the NP, and thereby relative drug distribution, to the tumor cells themselves 49, the presence of stroma and blood vessels were shown to be important for the delivery and retention of NP in the whole tumor. Previous work has indicated that supported blood vessels are required for optimal delivery 50. In each of the three models, the ratio of AZD2811 in the stroma to tumor compartments remained consistent for at least 10 days post-dosing. As the NP-formulation slowly releases AZD2811 1, the high stromal NP may act as a drug depot sustaining exposure in the tumor tissue. MSI showed that AZD2811 is released and diffuses away from the NP as AZD2811 N-desethyl metabolite. The local drug release can potentially provide additional anti-tumor benefit beyond the peripheral exposure of AZD2811. Therefore, understanding the balance of drug delivered by peripheral and intra-tumoral release is important in dissecting the drivers of nanomedicine efficacy.

Our analysis also revealed that AZD2811 delivered via NP distributes preferably to regions that are high in macrophages. Whether this co-localization is coincidental, or whether macrophages play an active role in modulating AZD2811-NP distribution, warrants further investigation. While stromal and highly vascularized regions tend to have higher numbers of macrophages, macrophages or tumor associated macrophages (TAMs) that are present in systemic circulation and accumulate NPs may also actively transport NP into the tumor. It has been shown in the high-macrophage-containing 4T1 model, that depleting macrophages with a liposomal clondronate reduced PLGA-PEG NP uptake and efficacy, illustrating the importance of macrophages for tumor accumulation for a similarly sized 100 nm PEGylated polymeric NP 43. This accumulation of NP in TAMs may drive a local drug reservoir or depot. Intra-vital microscopy suggested following accumulation of NP in TAMs, drug re-distributed from TAMs with increased free drug accumulating in tumor cells 43. A separate study of smaller fluorescently labelled ~65 nm PEG-b-pHPMA-based core-crosslinked polymeric micelles in 4T1 tumors showed 33% of particles were taken up intracellularly, the majority by phagocytic immune cells, while only 1.5% of the NPs were present in tumor cells after 48 h 42. The limitation of these studies is that they have been performed in macrophage-enriched 4T1 tumors, and the phenomenon has not been validated in other more clinically relevant models.

For many years the nanomedicine field has grappled with concepts like the EPR effect which are difficult to model and provide direct evidence to support conclusions. At the same time, it is important to consider the effect of heterogeneity in the spatial distribution of NPs and their associated cargo on the efficacy of the treatment. The MSI-IMC approach enables these concepts to be examined across tumors at a throughput level that drives robust conclusions.

Coupling MSI and IMC to produce a multi-modal imaging approach provides improved granularity on nanomedicine-delivered drug distribution within the tumor microenvironment. No other approach currently enables a direct comparison of the distribution and relative quantification of drug and metabolite within the spatial localization of 30 different cell types or biomarkers *in situ* in clinically relevant animal tumor models, allowing the visualization of the drug distribution on the same tissue section as a detailed characterization of the TME. Data co-registration and full integration allows for spatially resolved inter-modality correlation analysis and deep insights into tissue composition in areas of high and low drug distribution. Moreover, this approach can be employed at a scale that allows comparison between models with different physical features to be performed. Finally, because IMC and MSI can be performed with different nanomedicines, it ultimately allows both targeted and untargeted platforms to be assessed for efficiency of delivery, and novel technologies to be compared directly with established nanomedicines. Understanding the relative distribution of both carrier and drug for different modalities in the context of complex TMEs over time and between doses enables new insights into the local-regional effects of nanomedicines on drug distribution which has been a challenge in the field for many years.

## Conclusions

We believe that integrated molecular imaging methods, as presented here, will be critical to the development of novel nanotherapeutics, understanding associated distribution patterns and efficacy for successful patient stratification. Investigating this systematically requires a broad model analysis employing a single technique with sufficient throughput capacity and data granularity. MSI can be performed with the throughput required to analyze multiple tumors within a timeline suitable to support a drug development program, including analysis on human samples. This knowledge influences the interpretation of the different mechanisms via which the nanomedicine may be delivering an anti-tumor effect within the mouse or patient.

## Supplementary Material

Supplementary methods, figures and tables.

## Figures and Tables

**Table 1:** Antibody panel used for imaging mass cytometry analysis. More information can be found in Table S5

Target	Cell marker	Metal tag
αSMA	Fibroblasts, Endothelial cells	141Pr
Vimentin	Mesenchymal cells	143Nd
Collagen 1*	Extracellular matrix, stroma	144Nd
CD68*	Monocyte lineage	145Nd
Cleaved Caspase 3*	Apoptosis	147Sm
Pan-CK	Epithelial cells	148Nd
Ly6G	Neutrophils	151Eu
desmin*	Muscle cells	152Sm
CD11c*	Dendritic cells (DCs)	153Eu
CD11b	Myeloid lineage	154Sm
F4/80*	Macrophages	155Gd
CD163*	M2 macrophages	156Gd
E-Cadherin	Epithelial cells	158Gd
pNDRG1*	Glucose metabolism	159Tb
GLUT1*	Hypoxia	160Gd
pAMPK*	AMPK activation	162Dy
CD31	Endothelial cells	165Ho
EpCam (CD326)	Epithelial cells	166Er
Ki67	Proliferation	168Er
CD206	M2 macrophages	169Tm
Arg1*	M2 macrophages	170Er
pS6	mTOR activation	172Yb
TTF1*	Transcription Factor	173Yb
γH2AX*	DNA damage	173Yb
MHCII (I-A/I-E)	Myeloid polarization (M1 macs, DCs)	174Yb
CD45	Leukocyte marker	175Lu
pHH3	Mitosis (PD marker)	176Yb

*Antibodies marked with asterix were custom metal-labelled in-house. More information can be found in the Supplementary Material.
