# Supplementary material for: Multi-modal molecular imaging maps the correlation between tumor microenvironments and nanomedicine distribution
Source: Theranostics. 2022 Feb 14;12(5):2162–74. doi: 10.7150/thno.68000 (PMC8899579; doi:10.7150/thno.68000)
Supplement: Supplementary file 1 — Supplementary methods, figures and tables. [file thnov12p2162s1.pdf]

Supporting Information

Multi-modal molecular imaging maps the correlation between tumor microenvironments and nanomedicine distribution

Nicole Strittmatter[1,2], Jennifer I. Moss[3], Alan M. Race[4], Daniel Sutton[1], Jaime Rodriguez Canales[5], Stephanie Ling[1], Edmond Wong[6], Joanne Wilson[7], Aaron Smith[7], Colin Howes[7], Josephine Bunch[8], Simon T. Barry[3^], Richard J.A. Goodwin[1,9✉^], Marianne B. Ashford[10,✉^]

1 Imaging and Data Analytics, Clinical Pharmacology & Safety Sciences, R&D, AstraZeneca, Cambridge, UK
2 Department of Chemistry, Technical University of Munich, Garching, Germany
3 Bioscience, Discovery, Oncology R&D, AstraZeneca, Cambridge, UK
4 Institute of Medical Bioinformatics and Biostatistics, University of Marburg, Germany
5 Translational Pathology & Biomarker Analysis, Translational Medicine, R&D Oncology, AstraZeneca, Gaithersburg, MD, USA
6 Antibody Discovery and Protein Engineering (ADPE), R&D, AstraZeneca, Cambridge, UK
7 DMPK, Oncology R&D, AstraZeneca, Cambridge, UK
8 National Centre of Excellence in Mass Spectrometry Imaging (NiCE-MSI), National Physical Laboratory, Teddington, UK
9 Institute of Infection, Immunity and Inflammation, College of Medical, Veterinary and Life Sciences, University of Glasgow, Glasgow, UK
10 Advanced Drug Delivery, Pharmaceutical Sciences, R&D, AstraZeneca, Macclesfield UK

^authors jointly supervised this research

Corresponding authors:

✉Marianne B. Ashford, Pharmaceutical Sciences, R&D, AstraZeneca, Macclesfield, UK. marianne.ashford@astrazeneca.com
✉Richard J.A. Goodwin, Clinical Pharmacology & Safety Sciences , R&D, AstraZeneca, Cambridge, UK. richard.goodwin@astrazeneca.com

Summary: Extended Materials and Methods on drugs and PDX models used, further instrument parameters for LC-MS and DESI-MSI analyses, IMC antibody conjugation and ROI selection, and advanced image analysis (H&E tissue classification, MSI and IMC co-registration and integrative analysis). Additional results on correlation analysis of AZD2811 with IMC features and tumor tissue

subtypes, additional drug localization images at identical time points, additional bioanalysis results and % ID/g per model and tissue type.

Extended Methods and Materials

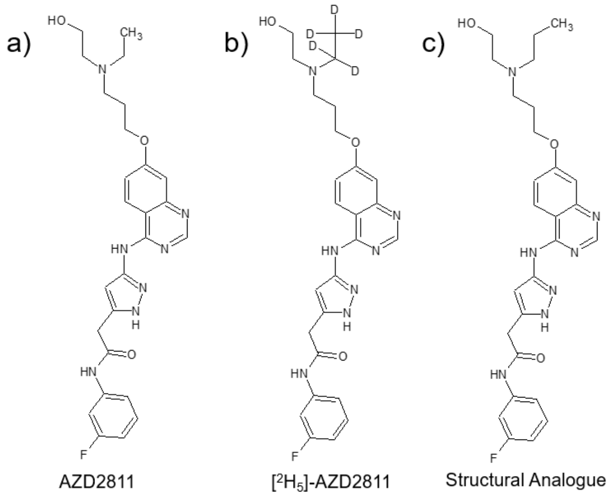

Figure S1. Molecular structures of drugs used in this study. a) NP-delivered AZD2811, b) intravenously dosed free drug [$^2H_5$]-AZD2811, c) structural analogue used for normalization purposes.

Table S1. Properties of the PDX models used in this study.

Tumor Designation	Tumor Number	Pre-Implantation Chemo/Radiotherapy	Stage at Implantation	Histology	Primary/ Metastasis/ Recurrent	Site of origin	Differentiation	Patient Age at Surgery	Gender	IT (d) median	TR (%) median
CXF	1297	no	M1 Liver	adeno carcinoma	Metastasis	Liver	poor	61	male	27 (23-42)	80 (67-89)
LXFE	2257	not available	pT3 pN0(0/24) L0V0	squamous cell carcinoma	Primary	Lung	moderate	73	male	25	
OVXF	899	no	T3N0M0	serous adeno carcinoma	Primary	ovary	good	76	female	24 (14-36)	67 (47-94)

These models were derived from surgical specimens of primary or metastatic patient tumors and were passaged in nude mice until stable growth characteristics were established.

LC-MS calibration curves

A)

Compound name: AZ11792866
Correlation coefficient: r = 0.989454, r^2 = 0.979020
Calibration curve: 0.0015789 * x + 0.00260688
Response type: Internal Std (Ref 4), Area * (IS Conc. / IS Area)
Curve type: Linear, Origin: Exclude, Weighting: 1/x^2, Axis trans: None

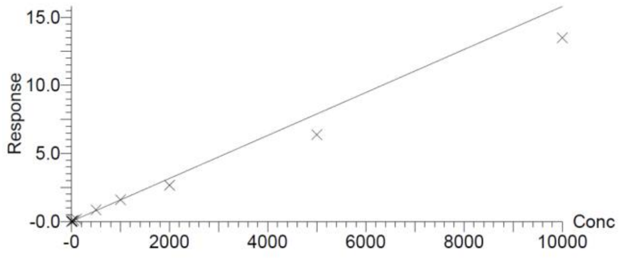

B)

Compound name: AZ12102238
Correlation coefficient: r = 0.993665, r^2 = 0.987370
Calibration curve: 0.000112675 * x + 0.000294182
Response type: Internal Std (Ref 4), Area * (IS Conc. / IS Area)
Curve type: Linear, Origin: Exclude, Weighting: 1/x^2, Axis trans: None

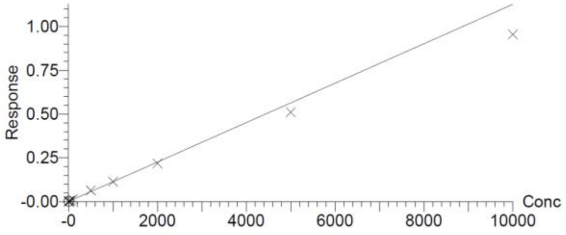

Figure S2. LC-MS calibration curves for AZD2811 (A) and metabolite (B).

Table S2. Mass Spectrometer and UPLC system parameters for bioanalysis

**Mass Spec**	Waters Xevo TQS		
**UPLC system**	Waters Acquity i-Class		
**Column**	Phenomenex Kinetix C18 50 x 2.1, 1.7 µm		
**Solvent A**	95% Water, 5% MeOH + 0.1% Formic acid		
**Solvent B**	95% MeOH, 5% Water + 0.1% Formic acid		
**Gradient**	Time (min)	**%A**	**%B**
	0	95	5
	0.3	95	5
	2.2	5	95
	2.6	5	95
	2.61	95	5
	2.8	95	5
**Flow**	0.6 mL/min		
**Run time**	2.8 min, use a divert valve for initial 0.5 min		

Table S3. Optimization parameters for mass spectrometry analysis for bioanalysis

Compound	Ionization mode	Polarity	Parent ion	Daughter ion	Cone voltage (V)	Collison Energy	Retention Time (min)
AZD2811	ESI	Positive	508.233	130.177	10	22	1.24
AZD2811 d5	ESI	Positive	513.150	135.216	20	22	1.24
AZ12102238	ESI	Positive	415.177	130.215	60	22	0.79
AZ10024306 (ISTD)	ESI	Positive	408.253	174.189	20	22	1.69

DESI-MSI

Table S4. Instrumental parameters for DESI-MSI analysis.

Parameter	Setting
**Polarity**	Negative
**Mass Range**	*m*/*z* 250-1000
**AGC Target**	5E6 (AGC off)
**Injection Time**	250 ms
**Resolution**	70,000 at *m*/*z* 200
**S-Lens RF Potential**	100
**Spray Voltage**	-4.5 kV
**Capillary Temperature**	320 °C
**Distance Sprayer - Sample**	1.5 mm
**Angle Sprayer - Sample**	80°
**Distance Sprayer - MS inlet**	7 mm
**Angle MS inlet - Sample**	10°
**Distance MS inlet - Sample**	<<1 mm
**Solvent**	MeOH/H_2O (95:5 *v*/*v*)
**Solvent Flow rate**	1.0 µL/min
**Nebulizing gas pressure**	6.5 bar (N4.8 N_2)

Morphological tissue classification

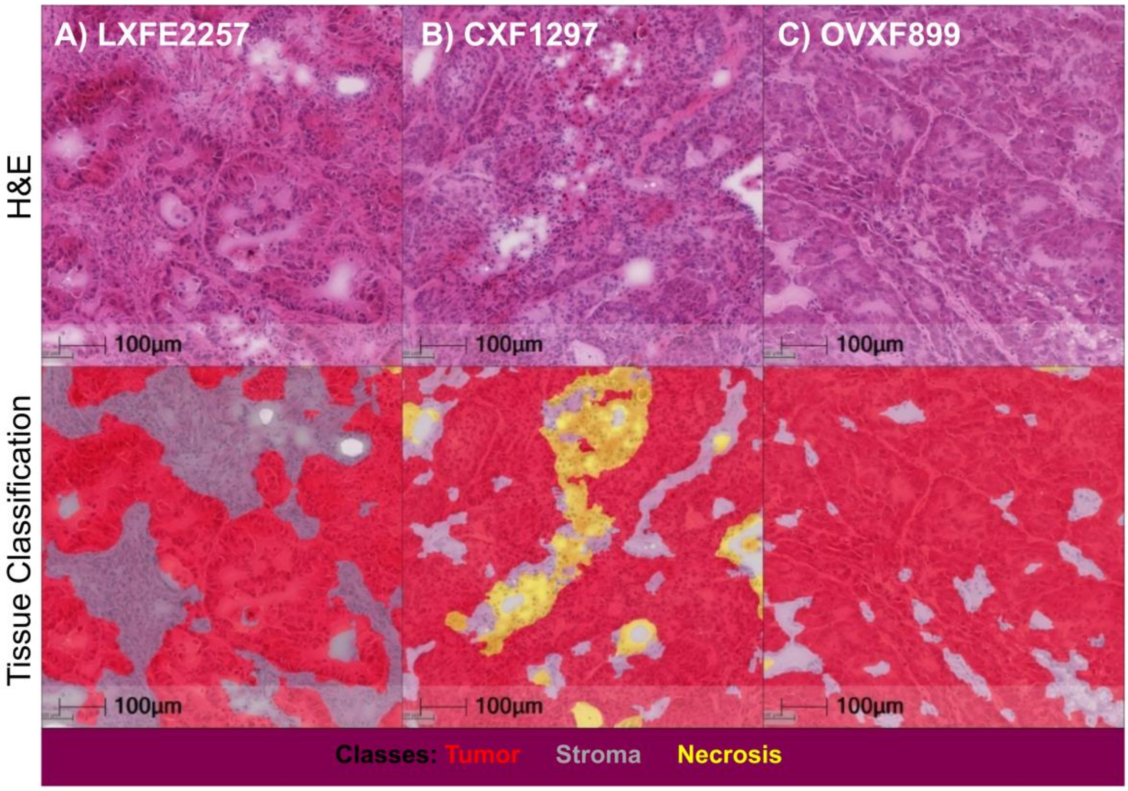

Figure S3. Top row: H&E for PDXs LXFE2257 (A), CXF1297 (B) and OVXF899 (C). Bottom row: Corresponding tissue classification into tumor (red), stroma (grey) and necrosis (yellow).

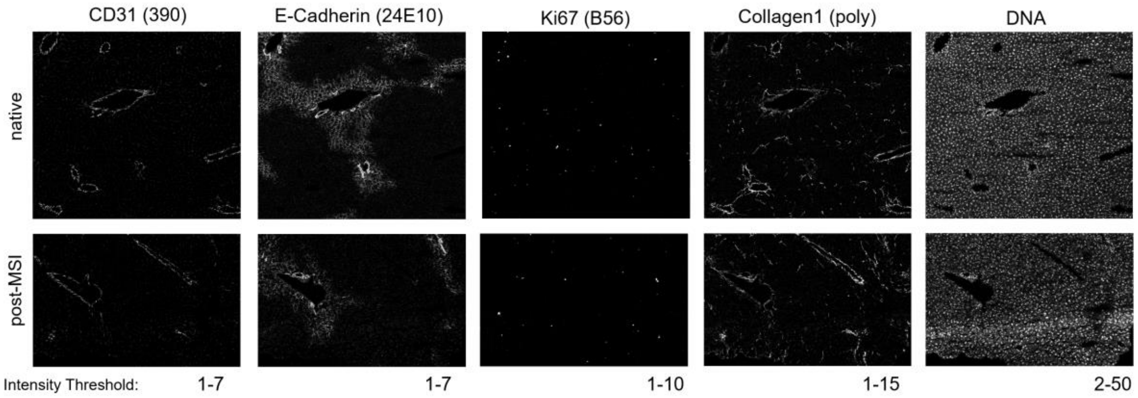

Figure S4. Comparison of IMC images obtained from native fresh frozen tissue sections as well as from tissues that were previously subjected to DESI-MSI analysis. Corresponding images were acquired within the same plasma strike and from the same glass slide (so underwent exact same staining procedure).

Morphological characterization of PDX models

Histological examination by a pathologist (JR-C) from all three PDX models showed malignant neoplasms with morphological features of carcinomas. These included tumor cells with recognizable cytoplasm, polygonal shape and variable moderate size, arranged in solid cohesive sheets and focal keratinization (lung model) or distorted glandular structures (ovarian and colon models). All PDX tumor models showed atypical cytological features including anisokaryosis and focal tumor necrosis. PDX models originated from ovarian and colon carcinomas showed morphological features concordant with adenocarcinomas ranging from moderate to poor degrees of histological differentiation, with tumor cells arranged in solid patterns and also around distorted luminal spaces, while the PDX model from lung cancer showed tumor cells arranged in solid sheets with bulging and infiltrative pattern of grow and signs of focal keratinization and necrosis concordant with squamous cell differentiation.

Imaging mass cytometry was next applied on ROIs of the same tissue section previously subjected to MSI analysis in order to identify tissue features associated with heterogenous AZD2811 distribution with higher granularity. A 29-plex panel of metal-tagged antibodies was developed, comprising markers highlighting the underlying tissue architecture (vasculature markers, stroma, tumor cells), complemented with several markers to visualize the immune cell landscape and several signaling/phenotype markers to further characterize certain cell subsets with regards to activation/polarization or metabolic state (see Table 1). By coupling the distribution patterns of the parent drug and the metabolites using DESI-MSI with the detailed characterization of the TME by IMC, it was possible to establish a high-resolution assessment of the distribution of the AZD2811 in specific regions of each tumor model.

High level review of the resulting IMC images reveals clear differences with regards to the amount and two-dimensional arrangement of the tumor-associated stroma in the three models (see Figure 1E-G). αSMA, a marker for fibroblasts and pericytes, presents as thick organized fibers in the lung model (Figure 1E), while a similar fiber arrangement was not observed in the colon or ovarian tissues. Percent positive cell populations were obtained over the entire ROI and the tissue classified as tumor cells only.

Figure 1H+I show markers which are more meaningful on a whole tissue section level, such as those characterizing the vasculature system or extracellular space, while Figure 1J shows markers which are expected to be predominantly

expressed in the tumor cell compartment. The ovarian model presents highest in vimentin (mesenchymal cells) and lowest in E-cadherin (epithelial cells), whereas the colon model is high in E-cadherin and lowest with regards to vimentin expression. The lung model on the other hand is strongly expressing both markers. On the epithelial-mesenchymal spectrum (as represented by the relative amount of E-cadherin and vimentin), the colon model is on the most epithelial with minimal mesenchymal component while the ovarian model is the most mesenchymal with little epithelial component. Pan-CK expression, representing epithelial cells, is decreasing from colon, ovarian to lung models. None of the tumor cells was found to express Ep-CAM, the epithelial cell adhesion molecule. A comparable amount of blood vessels (CD31, endothelial cells) and desmin (muscle cells) was observed in all three models. Lung presents with the highest collagen 1 (extra cellular matrix, ECM) and αSMA proportion, while collagen 1 is similar for colon and ovarian models.

Summary IMC antibody staining procedure.

Slides were fixed in 4% paraformaldehyde (PFA), washed, permeabilized for 5mins using 1× casein solution containing 0.1% Triton X-100, washed, blocking solution was applied for 30mins, washed, antibody panel (Table S5) was applied and incubated at 4ºC overnight, washed, DNA Ir-intercalator (Fluidigm, diluted 1:400 in PBS) was applied for 30mins, washed, the slide was eventually dipped in deionized water and dried. All washing steps consisted of 3x 5mins in fresh PBS. Unless stated otherwise, all steps were performed at room temperature.

Summary custom IMC antibody generation

Custom antibodies were labelled according to the manufacturer's instruction. To summarize, antibodies were reduced, and buffer exchanged into the recommended buffers in 0.5 mL spin concentrators. Where applicable, lyophilized antibodies were rehydrated with PBS, washed 3 times in 0.5 mL spin concentrators with PBS before reduction and washing according to the manufacturer's instruction. Separately, MAXPAR polymer was rehydrated then incubated with Lanthanide metal before removing excess metals and buffer change. Reduced antibody and Lanthanide metal bound polymer were mixed and incubated for 1.5 h at 37 °C before washing and storage as recommended.

Table S5. More details on antibody panel shown in Table 1.

Target	Clone	Metal tag	Product code	Dilution
αSMA	1a4	141Pr	3141017d	75
Vimentin	D21H3	143Nd	3143027D	100
Collagen 1*	Polyclonal	144Nd	AB758	200
CD68*	FA-11	145Nd	MCA1957GA	100
Cleaved Caspase 3*	E83-77	147Sm	ab208003	50
Pan-CK	C11	148Nd	3148020D	100
Ly6G	1A8	151Eu	3151010B	50
desmin*	Poly	152Sm	AF3844	100
CD11c*	D1V9Y	153Eu	#97585	50
CD11b	M1/70	154Sm	3154006B	50
F4/80*	CI:A3-1	155Gd	MCA497GA	100
CD163*	TNKUPJ	156Gd	14-1631-82	50
E-Cadherin	2.4E+11	158Gd	3158029D	100
pNDRG1*	D98G11	159Tb	#5482	100
GLUT1*	EPR3915	160Gd	ab196357	50
INOS (devalidated)	CXFNT	161Dy	3161011B	50
pAMPK*	40H9	162Dy	#2535	50
CD31	390	165Ho	3165013B	100
EpCam (CD326)	G8.8	166Er	3166014B	100
αSMA* (excluded)	Polyclonal	167Er	ab5694	100
Ki67	B56	168Er	3168001B	100
CD206	CD68C2	169Tm	3169021B	50
Arg1*	Polyclonal	170Er	AF5868	50
pS6	N7-548	172Yb	3172008A	100
TTF1*	EP1584Y	173Yb	ab216648	100
γH2AX*	JBW301	173Yb	05-636	50
MHCII (I-A/I-E)	M5/114.15.2	174Yb	3174003B	50
CD45	30-F11	175Lu	3175010B	100
pHH3	HTA28	176Yb	3176024D	200

*custom antibodies

More details on MSI and IMC Co-registration and correlation analysis

Tissue and background were separated in the MSI data using *k*-means clustering. Peak detection (with a maximum of the top 1000 peaks detected, with a ppm tolerance of 5 ppm) was performed using SpectralAnalysis.[22] *k*-means clustering (k = 2) was performed on the L2 normalized peak reduced datacube to separate background and tissue regions.

Representative images were generated for both modalities for the multimodal registration process. For IMC, a representative image of the slide was generated from ‘panorama images’ (optical images of the slide acquired prior to experiment setup, which were extracted from the instrument format along with the relative slide coordinates from where the image was acquired) and the measured DNA marker (placed using relative slide coordinates). For MSI, the ion image *m/z* 885.5499 (+/- 3 ppm) was used as this showed tissue boundaries (and some tissue features) clearly. Multimodal registration was performed manually using the representative images to optimize an affine transform. Individual transformation matrices for each IMC region were computed by combining the affine transform between representative images and the transform matrix for converting the IMC acquisition region to the representative slide image.

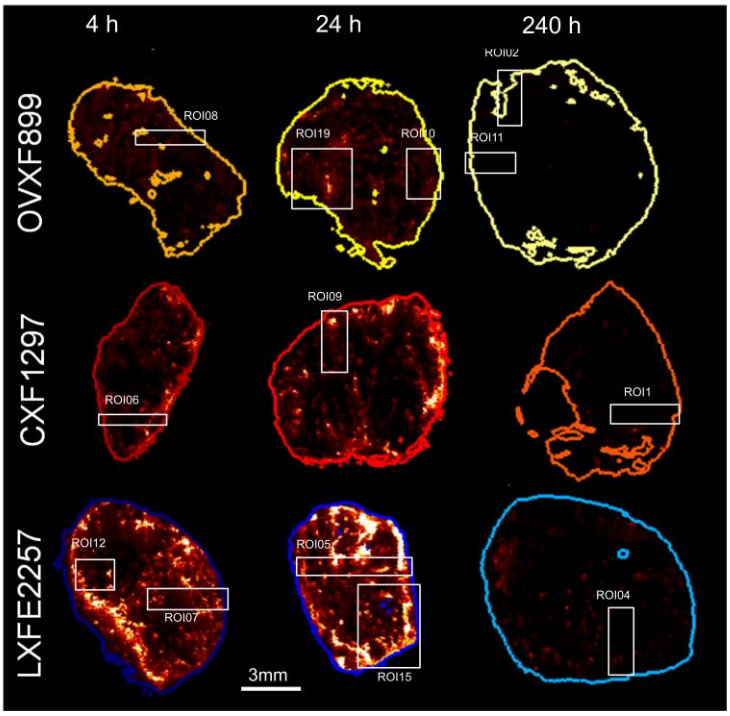

Figure S5. Drug distribution over all analyzed tissue sections. IMC regions of interest (ROIs) are indicated by white boxes and were chosen to incorporate both areas of high and low drug exposure.

Individual cell data (acquired by applying cell segmentation to IMC data) were transformed to the MSI space by transforming the cell location in the IMC data using the respective transform generated above. The number of cells with a given IMC marker or combination of markers (phenotype) were counted per MSI pixel. Pearson correlation was then performed between the number of cells with a given marker (or combination of markers) and ion images.

Tissue classification in MSI

Tumor classification based on IMC-derived training set

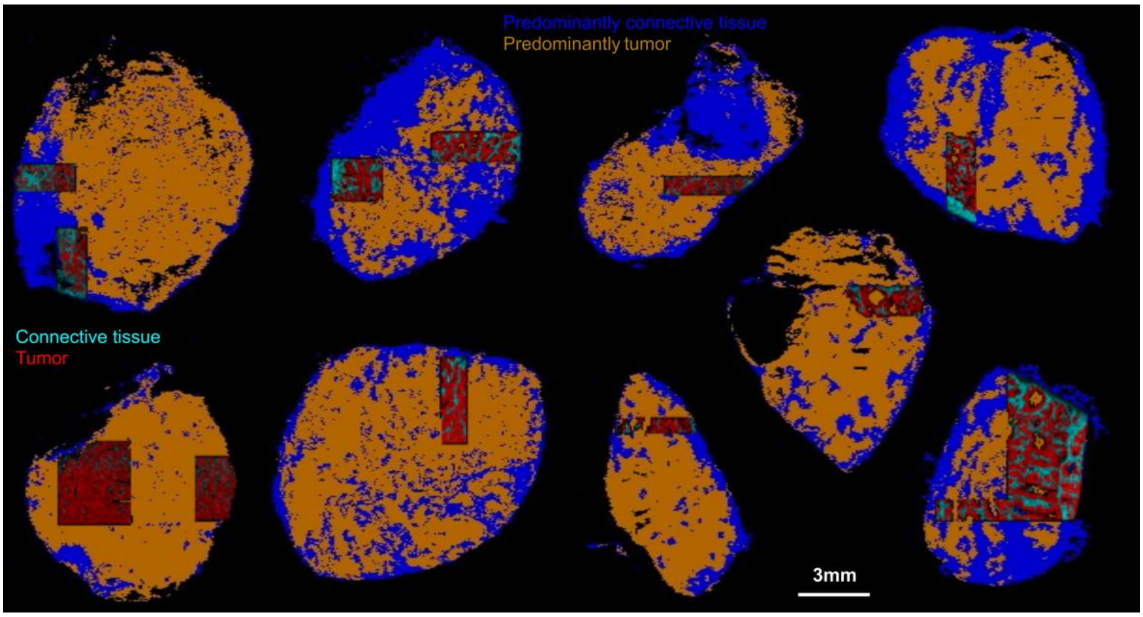

Figure S6. IMC training regions (red – tumor, cyan – connective tissue) used to classify DESI-MSI dataset into tumor (orange) and connective tissue (blue).

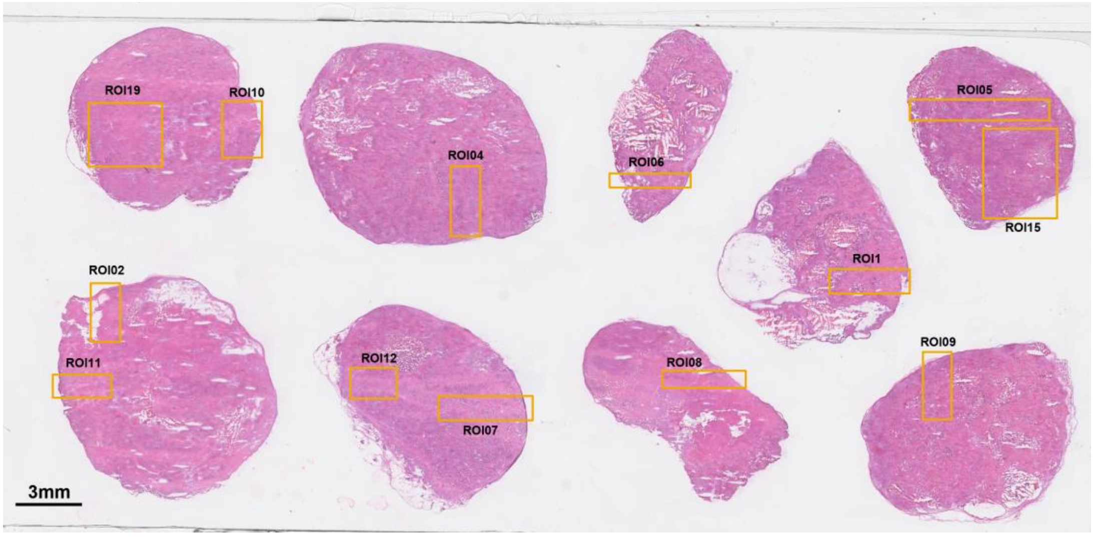

Figure S7. Corresponding H&E slide to data shown in Figure S4. Orange boxes indicate regions of interest (ROIs) chosen for IMC analysis.

Bioanalysis results

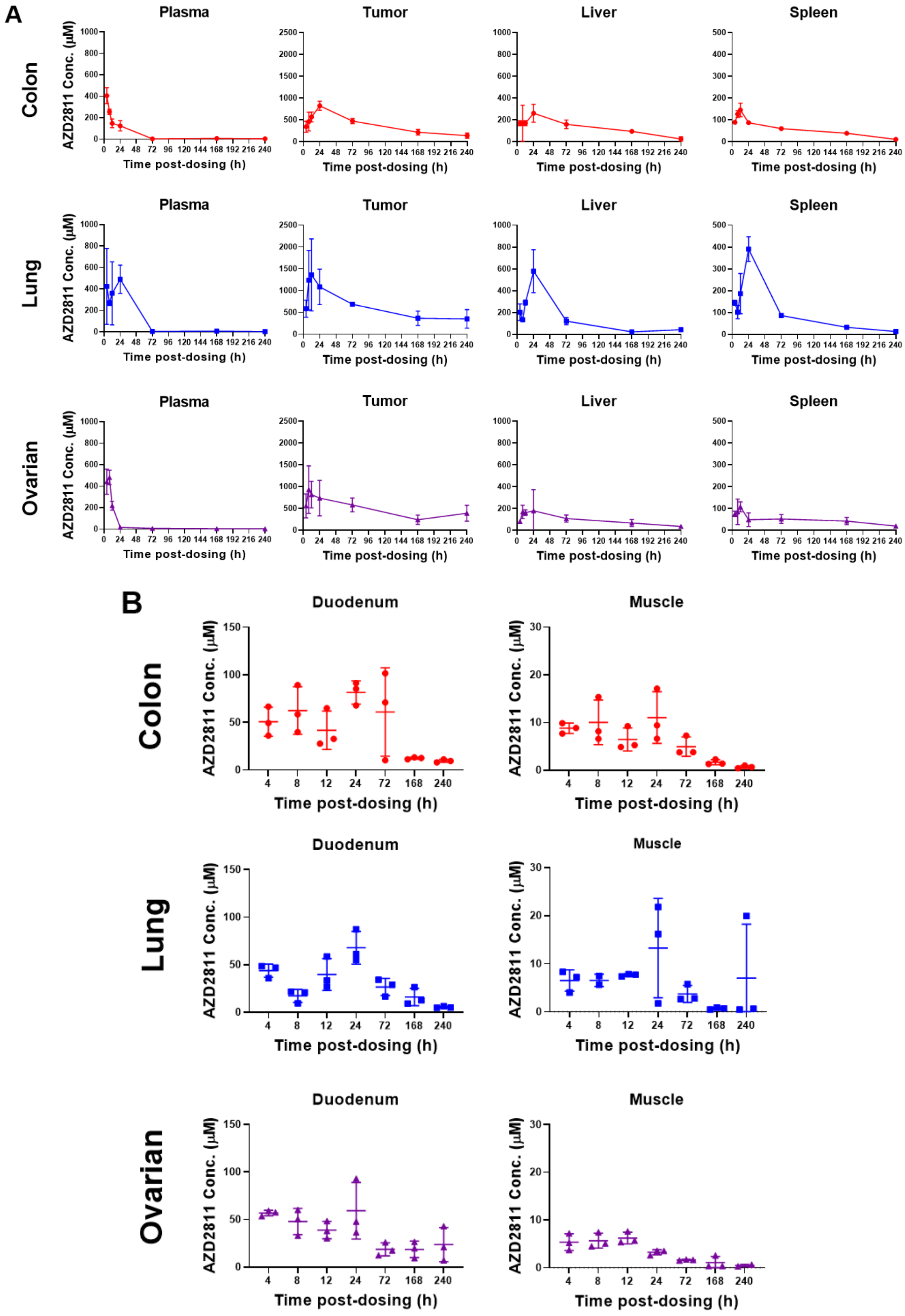

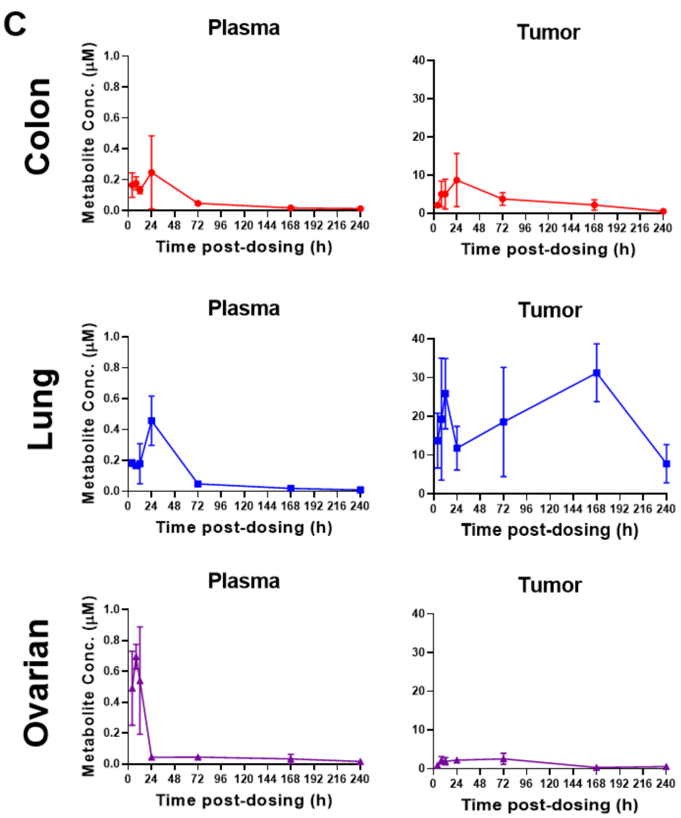

Figure S8. A) Plasma, tumor and tissue concentration of AZD2811 over time in mice bearing PDX tumors. B) Tissue concentration of AZD2811 over time in duodenum and muscle in mice bearing PDX tumors. C) AZD2811 metabolite concentration over time in plasma and tumor over time in mice bearing PDX tumors. Data expressed as mean +/- SD from n = 3 mice per time point.

Table S6. Percent of injected dose of AZD2811 per gram (% ID/g) of tissue at steady state in mice bearing PDX tumors.

	Colon	Ovarian	Lung
Tumor	9.2	6.1	8.0
Spleen	6.3	6.3	9.8
Liver	13.5	11.4	15.3
Muscle	0.48	0.20	0.39
Duodenum	4.3	2.9	3.1

Distribution of AZD2811-NP and [$^2H_5$]-AZD2811 in tumor at different time points

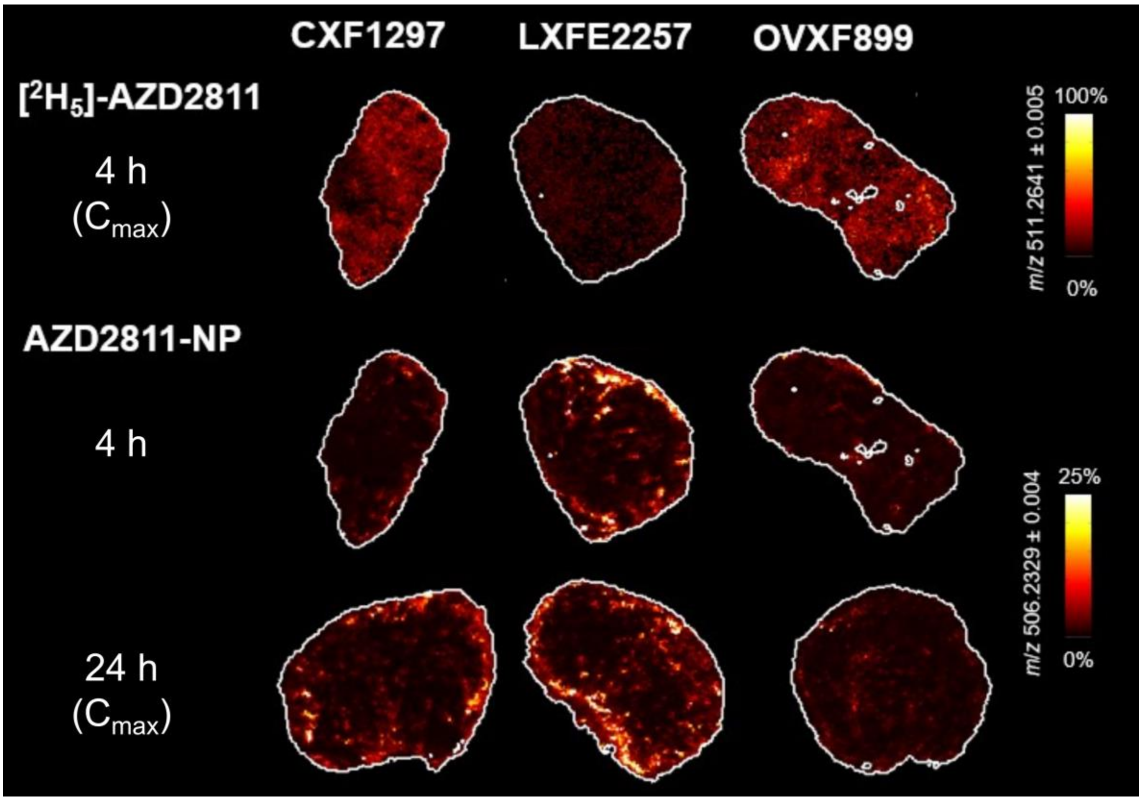

Figure S9. Distribution of [$^2H_5$]-AZD2811 (IV) dosed at 4 h and AZD2811 at 4 h and 24 h post-dose for colon (CXF1297), lung (LXFE2257) and ovarian (OVXF899) PDX models. Both drug images were normalized to *m/z* 520.2492 ± 0.005 (structural analogue).

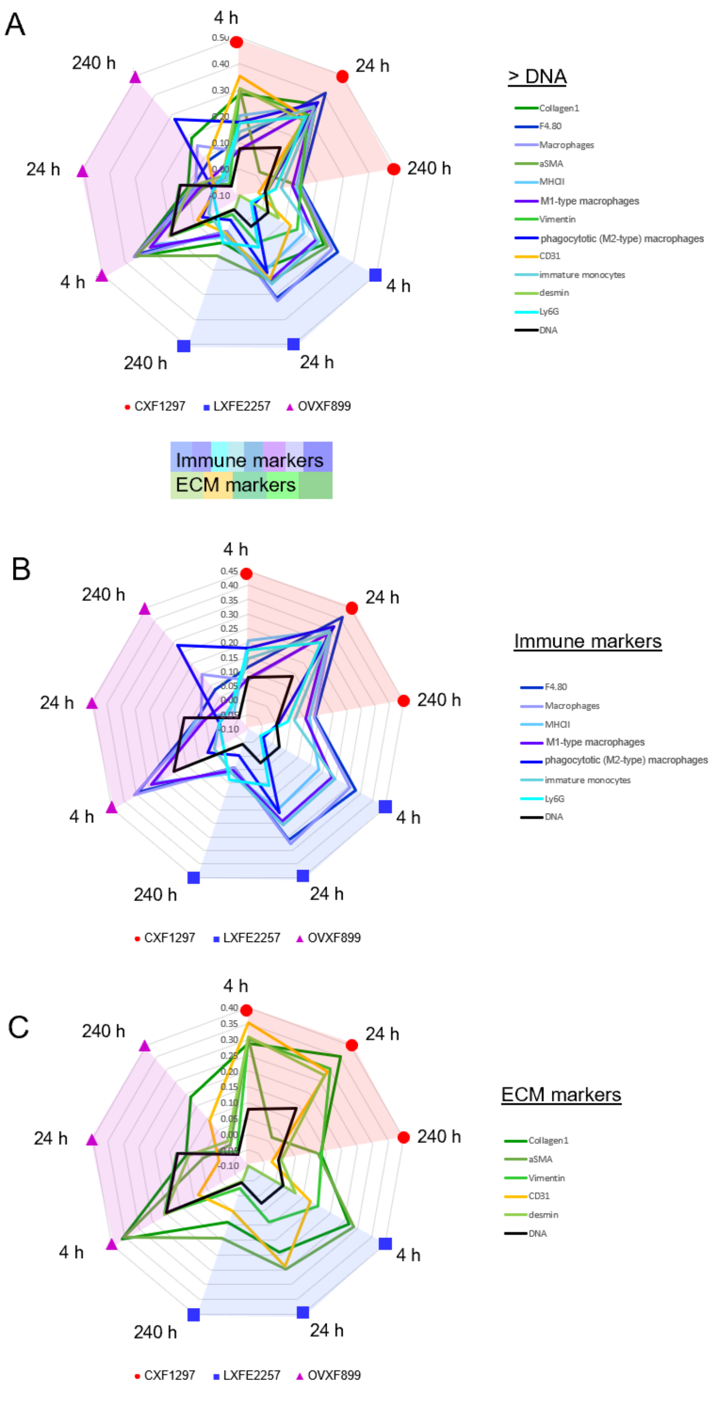

Figure S10. a) Radar plot showing correlation values between AZD2811-NP (DESI-MSI) and those IMC markers which show stronger correlation than DNA (that is a stronger correlation than the simple presence of a cell). b) and c) show correlations for the subset of markers corresponding to immune cell markers and extracellular matrix (ECM) markers only, respectively. Correlations were obtained between two images of different modalities and different spatial resolutions (50 µm x75 µm vs 1 µmx1 µm) and as such should not be viewed in absolute numbers but in relation to each other. Data presented in Table S7.

Table S7. Pearson Correlation values between AZD2811-NP (by DESI-MSI) and IMC single markers and multi-marker combinations (phenotypes). Conditional formatting applied: +1 (red), 0 (white), -1 (blue).

Model<br>Time point	Colon<br>4 h	Colon<br>24 h	Colon<br>240 h	Lung<br>4 h	Lung<br>24 h	Lung<br>240 h	Ovarian<br>4 h	Ovarian<br>24 h	Ovarian<br>240 h
Collagen1	0.29	0.35	0.13	0.27	0.19	0.09	0.36	0.10	0.18
F4/80	0.11	0.41	0.14	0.33	0.31	0.04	0.34	0.08	0.08
Macrophages	0.07	0.35	0.13	0.31	0.33	0.04	0.36	0.07	0.15
αSMA	0.31	0.02	0.12	0.28	0.25	0.14	0.35	0.04	-0.01
MHCII	0.21	0.35	0.12	0.18	0.20	0.07	0.27	0.08	-0.02
M1-type macrophages	0.08	0.33	0.10	0.24	0.24	0.05	0.29	0.06	0.02
Vimentin	0.30	0.30	0.14	0.15	0.09	-0.02	0.20	0.13	-0.04
Phagocytotic M2-type macrophages	0.18	0.36	0.01	-0.04	0.21	0.00	0.06	0.00	0.28
CD31	0.35	0.29	-0.02	0.13	0.24	0.05	0.08	-0.01	0.09
Immature monocytes	0.15	0.34	0.06	0.25	0.26	0.06	0.04	0.00	-0.01
Desmin	0.31	0.27	0.01	0.07	-0.10	-0.05	0.21	0.09	0.00
Ly6G	0.18	0.29	0.04	-0.05	0.11	0.09	0.00	0.00	0.00
DNA	0.08	0.14	0.00	0.03	0.03	-0.04	0.20	0.13	-0.05
pS6	0.06	-0.04	-0.02	0.19	-0.05	0.01	0.34	0.05	-0.06
Dendritic cells	0.03	0.04	0.02	0.16	0.01	0.00	0.02	-0.04	0.00
Neutrophils	0.16	0.06	0.03	-0.13	0.01	0.08	-0.01	0.01	0.03
EpCam	0.02	0.01	0.08	0.01	-0.04	-0.01	-0.03	-0.01	-0.02
pHH3	-0.01	-0.01	-0.04	0.01	-0.03	0.00	0.00	0.02	0.05
TTF1	-0.04	-0.09	0.05	-0.02	-0.09	0.04	-0.01	0.02	-0.04
Ki67	0.03	-0.13	-0.13	-0.09	-0.13	-0.09	0.17	0.16	-0.08
Pan-CK	-0.10	-0.19	-0.10	-0.11	-0.01	-0.06	0.09	0.25	-0.10
γH2AX	-0.11	0.01	-0.04	-0.17	-0.10	-0.08	0.05	0.03	-0.06
pAMPK	0.03	-0.03	-0.06	-0.19	-0.13	-0.04	-0.01	0.00	-0.09
E- Cad	-0.05	-0.18	-0.11	-0.24	-0.18	-0.12	-0.01	0.02	-0.13
Glut1	-0.04	-0.19	-0.09	-0.27	-0.14	-0.11	-0.11	-0.13	-0.08
pNDRG1	-0.12	-0.14	-0.03	-0.30	-0.09	-0.17	-0.20	-0.18	-0.03

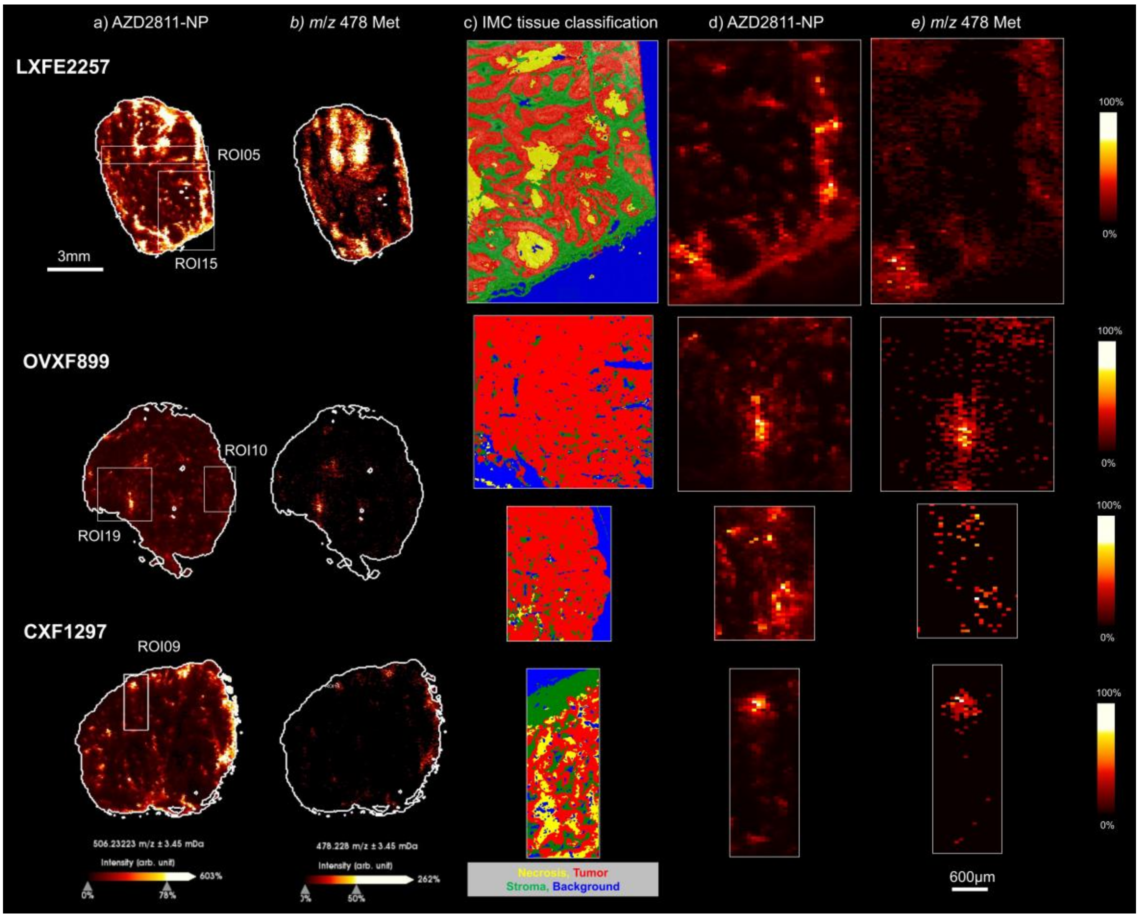

Figure S11. Comparison of the spatial distribution of drug and metabolite in relation to the TME at 24h (C_{max}) for all PDX models. (a-b) Whole section MSI images of drug (a) and AZD2811 Hydroxy-QPA-N-desethyl metabolite (detected at *m/z* 478) (b). Images shown are on the same intensity scale for all images of shown within either (a) or (b). (c) IMC random forest tissue classification map for ROI indicated in panel (a). (d-e) Zoom of MSI images of AZD2811-NP (d) and metabolite (e) in those areas that were subjected to IMC analysis. Each image in each (d-e) was intensity scaled to the highest pixel in the respective ROI to improve visibility. All tissues and ROI are in original size ratios.

Assessment of stromal composition in areas of high and low AZD2811

For this analysis, we used the largest ROIs recorded (for LXFE2257, these are covering 13.8mm^2 of the tissue area) and exhibit a sizeable proportion of stroma (38%, comprising 53773 cells) as well as showing some heterogeneity of drug distribution over the stroma tissue compartment. Large ROIs are important for this type of analysis to capture enough drug heterogeneity and cell types of low abundance such as some immune cell subtypes. We used the cell object data (each cell has a position and tissue classifier assigned) and binned it into pixels corresponding to the MSI data. This data was then divided into “drug high” vs.

“drug low” stroma pixels depending on whether the drug signal observed in each pixel lies within the top 50% or bottom 50% of pixels based on NP-delivered AZD2811 abundance. Cell types were summed for all “drug high” and “drug low” pixels to investigate compositional changes between those areas that show drug accumulation. Average drug abundance in drug high pixels is 5.5-fold increase over the intensity observed in drug low pixels.

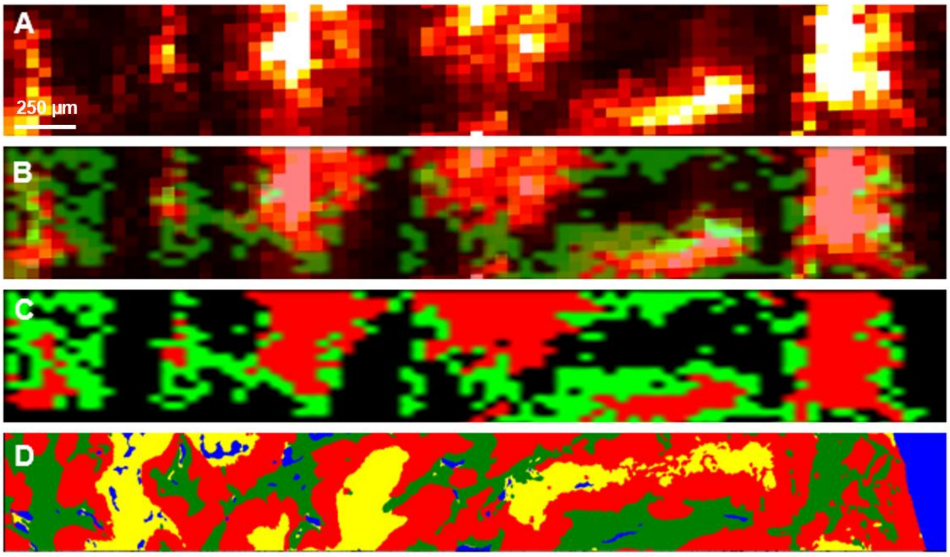

Figure S12. A) NP-delivered AZD2811 drug signal for LXFE2257 24 h (the smaller of both ROI) by DESI-MSI. B) Drug signal overlaid onto C. C) Stroma pixels from D classified into pixels of high (top 50%, red) and low (bottom 50%, green) of drug abundance. D) IMC tissue classifier used to identify stroma pixels. Panels A and D identical with those in Figure 4.
